# Inactivation of ornithine aminotransferase by (1*R*,4*S*)-4-Amino-3-(trifluoromethyl)cyclopent-2-ene-1-carboxylic acid via a stable quinonoid intermediate

**DOI:** 10.1007/s00044-026-03538-1

**Published:** 2026-03-15

**Authors:** Koon Mook Kang, Abigail L. Vargas, Wei Zhu, Inna Sokolenko, Dali Liu, Richard B. Silverman

**Affiliations:** 1 Department of Chemistry, Northwestern University, Evanston, IL USA; 2 Chemistry of Life Processes Institute, Northwestern University, Evanston, IL USA; 3 Department of Chemistry and Biochemistry, Loyola University Chicago, Chicago, IL USA; 4 Department of Molecular Biosciences, Northwestern University, Evanston, IL USA; 5 Center for Developmental Therapeutics, Northwestern University, Evanston, IL USA; 6 Department of Pharmacology, Feinberg School of Medicine, Northwestern University, Chicago, IL USA; 7 Present Address: Insilico Medicine Shanghai Ltd., Shanghai, China

**Keywords:** Ornithine aminotransferase, Pyridoxal 5’-phosphate-dependent enzyme, Hepatocellular carcinoma, Mechanism-based inactivator, Stable quinonoid intermediate, X-ray crystallography

## Abstract

Ornithine aminotransferase (OAT), a pyridoxal 5’-phosphate (PLP)-dependent enzyme, is a key contributor to glutamine supply in cancer cells, suggesting its therapeutic potential for hepatocellular carcinoma (HCC), the most common form of liver cancer. To identify an initial set of OAT inactivators, we have tested inactivators of γ-aminobutyric acid aminotransferase (GABA-AT), a homologous PLP-dependent enzyme, with human OAT (*h*OAT) and identified several co-inactivators. Among the active molecules, (1*R*,4*S*)-4-amino-3-(trifluoromethyl)cyclopent-2-ene-1-carboxylic acid (**2**) has not been thoroughly investigated for its time-dependent kinetics and mechanistic pathways with OAT. In this study, we evaluated the time-dependent inactivation of *h*OAT by **2** and investigated the underlying mechanism, primarily based on X-ray crystallography. The results demonstrated that **2** acts as a time-dependent OAT inactivator with an inactivation efficiency (*k*_inact_/*K*_I_ = 5.1 min^−1^mM^−1^) approximately 30-fold higher than that for GABA-AT (*k*_inact_/*K*_I_ = 0.17 min^−1^mM^−1^) and, notably, revealed an inactivation pathway that proceeds via a stable quinonoid intermediate, as evidenced by the UV-Vis spectroscopy.

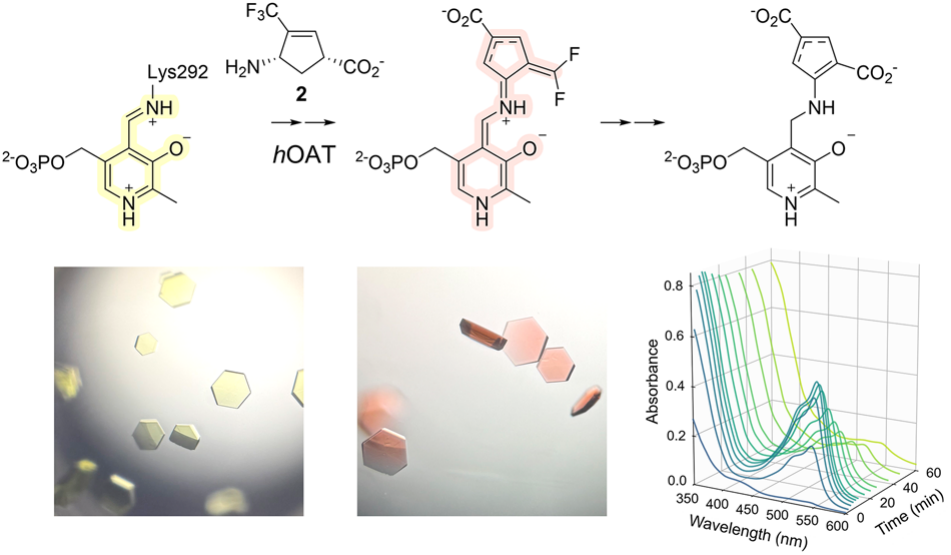

## Introduction

**Scheme 1:**
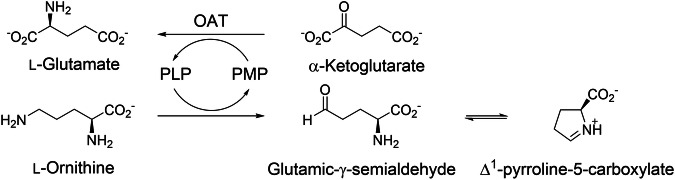
**Catalytic cycle of ornithine aminotransferase (OAT)**. PLP, pyridoxal 5’-phosphate; PMP, pyridoxamine 5’-phosphate

Over the past decade, ornithine aminotransferase (OAT), a pyridoxal 5’-phosphate (PLP)-dependent enzyme, has emerged as a therapeutic target for hepatocellular carcinoma (HCC) [1], which is the most common type of liver cancer, accounting for approximately 90% of cases [2]. OAT catalyzes the synthesis of glutamate from α-ketoglutarate (αKG) and pyridoxamine 5’-phosphate (PMP), as well as the PLP-dependent conversion of ornithine into glutamic-γ-semialdehyde (GSA), which spontaneously cyclizes to Δ^1^-pyrroline-5-carboxylate (P5C) (Scheme 1) [3]. In cooperation with glutamine synthetase (GS) and other metabolic enzymes, OAT contributes to the synthesis of glutamine, which is required in large amounts to support anabolic metabolism and rapid proliferation of cancer cells [1, 4]. Aberrant activation of the Wnt/β-catenin signaling pathway has been shown to act as an oncogenic driver in various cancers, including HCC [5]. Particularly, Wnt/β-catenin signaling has been reported to participate in important physiological processes in the liver cells [6], such as development [7, 8], growth [9, 10], regeneration [11], and metabolism [12]. Because Wnt/β-catenin signaling induces the expression of genes involved in glutamine metabolism in liver cells, which express OAT, GS, and the glutamate transporter GLT-1 protein [12, 13], OAT has been hypothesized to play a central role in supplying the glutamine that is excessively required by HCC cells and, therefore, suggested to be a potential therapeutic target for HCC. To support this, we have demonstrated that OAT inactivation suppresses the in vitro proliferation of HCC cells in our previous report [14].

**Fig. 1:**
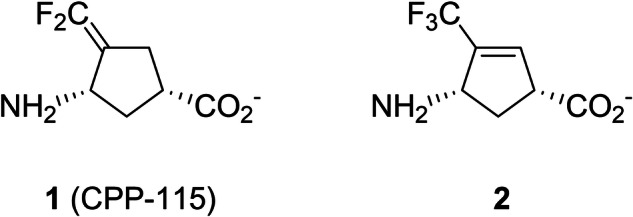
**Structures of CPP-115 (1) and (1*R*,4*S*)-4-amino-3-(trifluoromethyl)cyclopent-2-ene-1-carboxylic acid (2)**

**Scheme 2:**
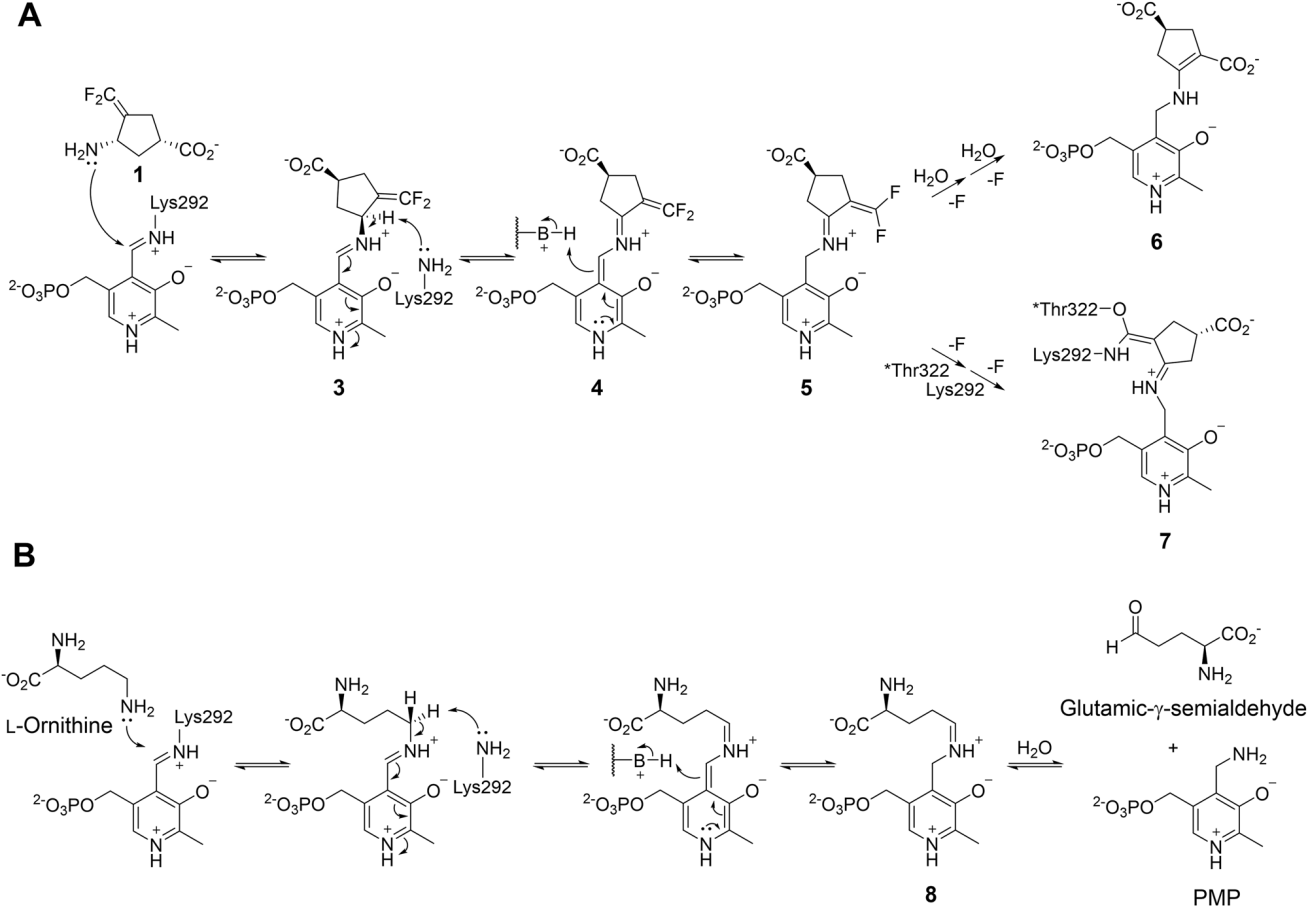
**Mechanisms of OAT**. (**A**) Inactivation mechanisms of OAT by **1**. (**B**) Mechanism of ornithine degradation by OAT

As OAT shares high similarity in substrate and active site structure with γ-aminobutyric aminotransferase (GABA-AT) [15, 16], which is in the same enzyme subgroup as OAT [17], we previously reported that several GABA-AT inactivators also inactivate human OAT (*h*OAT) [14]. We then investigated the inactivation mechanisms of these inactivators with *h*OAT [18**–**21] to understand their modes of action and leverage them for developing new OAT-selective inactivators. Among them, the OAT inactivation mechanism of CPP-115 (**1**; Fig. 1), a representative GABA-AT inactivator [22, 23], was proposed as described in Scheme 2A, based on the numerous biochemical studies, including X-ray crystallography [21]. As **1** was rationally designed as a mechanism-based inactivator (MBI) of GABA-AT, it follows the initial steps of the normal catalytic pathway of GABA upon binding in the GABA-AT active site [23], consistent with the definition of MBI [24, 25]. Similarly, **1** proceeds along the ornithine catalytic pathway with OAT [1, 15], undergoing formation of external aldimine (**3**) with PLP, followed by its γ-deprotonation, and subsequent re-protonation at the C4’ position of the resulting quinonoid intermediate (**4**) to yield the tautomerized ketimine (**5**) [21]. In contrast to the native ornithine degradation pathway (Scheme 2B), in which the corresponding tautomerized aldimine intermediate (**8**) is hydrolyzed to yield GSA, the δ-difluoromethylenyl group of **5** conjugates with the ketimine, thereby generating a Michael acceptor. This reactive intermediate is then attacked by a water molecule or by *Thr322 (asterisk indicates the adjacent subunit), leading to the formation of either a tight-binding adduct (**6**) or a covalent adduct (**7**) to irreversibly inactivate OAT (Scheme 2A). The elucidated inactivation mechanism has provided a basis for enhancing the GABA-AT/OAT selectivity of **1** [21, 26], exemplifying the use of detailed mechanistic insight toward fine-tuning the selectivity of MBIs and developing them as clinical candidates.

Among the reported OAT inactivators from our studies, (1*R*,4*S*)-4-amino-3-(trifluoromethyl)cyclopent-2-ene-1-carboxylic acid (**2**; Fig. 1) has been reported to inhibit *h*OAT with a *K*_i_ of 0.15 mM [14], while exhibiting only weak time-dependent inactivation of GABA-AT (*k*_inact_/*K*_I_ = 0.17 min^−1^mM^−1^) [27]. However, the time-dependent inactivation profile of OAT by **2** has not been determined. Hence, in this study, we evaluated the time-dependent inactivation of *h*OAT by **2** to compare its kinetic profile in *h*OAT with that of GABA-AT. We then investigated its inactivation mechanism for *h*OAT by a dialysis experiment, X-ray crystallography, and UV-Vis spectroscopy. Interestingly, it involves the observation of a stable quinonoid intermediate, which has not been reported with our other OAT inactivators, including another trifluoromethyl-containing molecule [18], cyclopentene [20, 28], and cyclohexene [29, 30] analogues.

## Results and discussion

### Time-dependent inactivation of *h*OAT and irreversibility

**Fig. 2:**
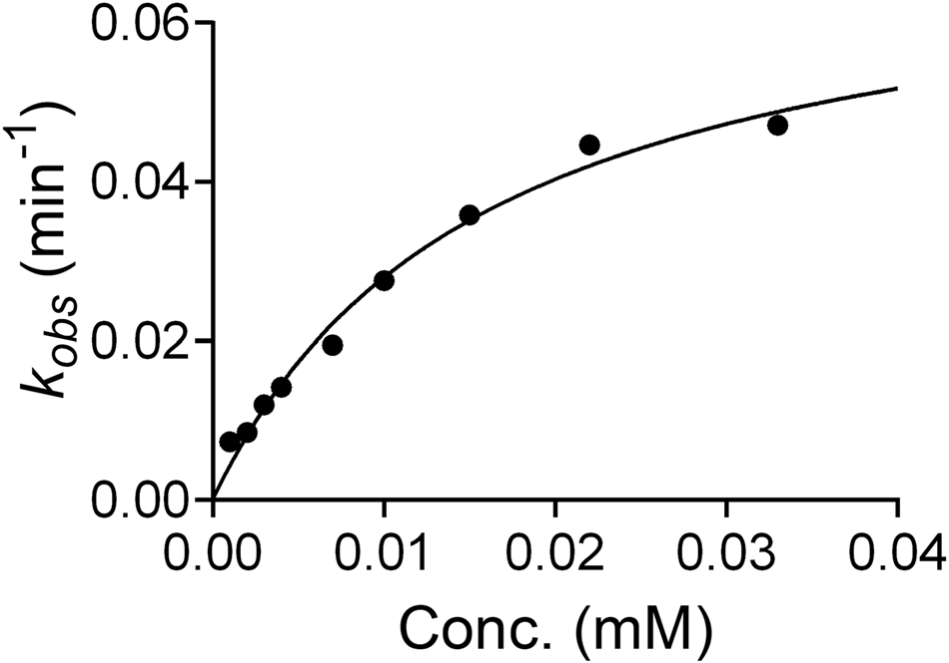
**Time-dependent assay results of 2 with** ***h*OAT**

To evaluate the time-dependent inactivation, we incubated **2** with recombinantly expressed *h*OAT, which was purified according to a previously reported protocol [31]. The residual enzymatic activity was measured at various concentrations of **2** and pre-incubation times, to calculate the observed inactivation rate constants (*k*_obs_) at each inactivator concentration. The concentration–*k*_obs_ plot (Fig. 2) was used to calculate the inactivation efficiency of the compound, which is defined as the ratio of the maximal inactivation rate (*k*_inact_) to the concentration for half-maximal inactivation rate (*K*_I_) [24], based on the previously reported protocol [32]. From the analysis, *k*_inact_, *K*_I_, and *k*_inact_/*K*_I_ were determined to be 0.062 min^−1^, 0.012 mM, and 5.1 min^−1^mM^−1^, respectively, indicating time-dependent inactivation of *h*OAT by **2**. When compared to the previously reported inactivation efficiency of **2** for GABA-AT (0.17 min^−1^mM^−1^) [27], the OAT/GABA-AT selectivity is 30, demonstrating that **2** acts as an OAT-selective inactivator [29].

**Fig. 3:**
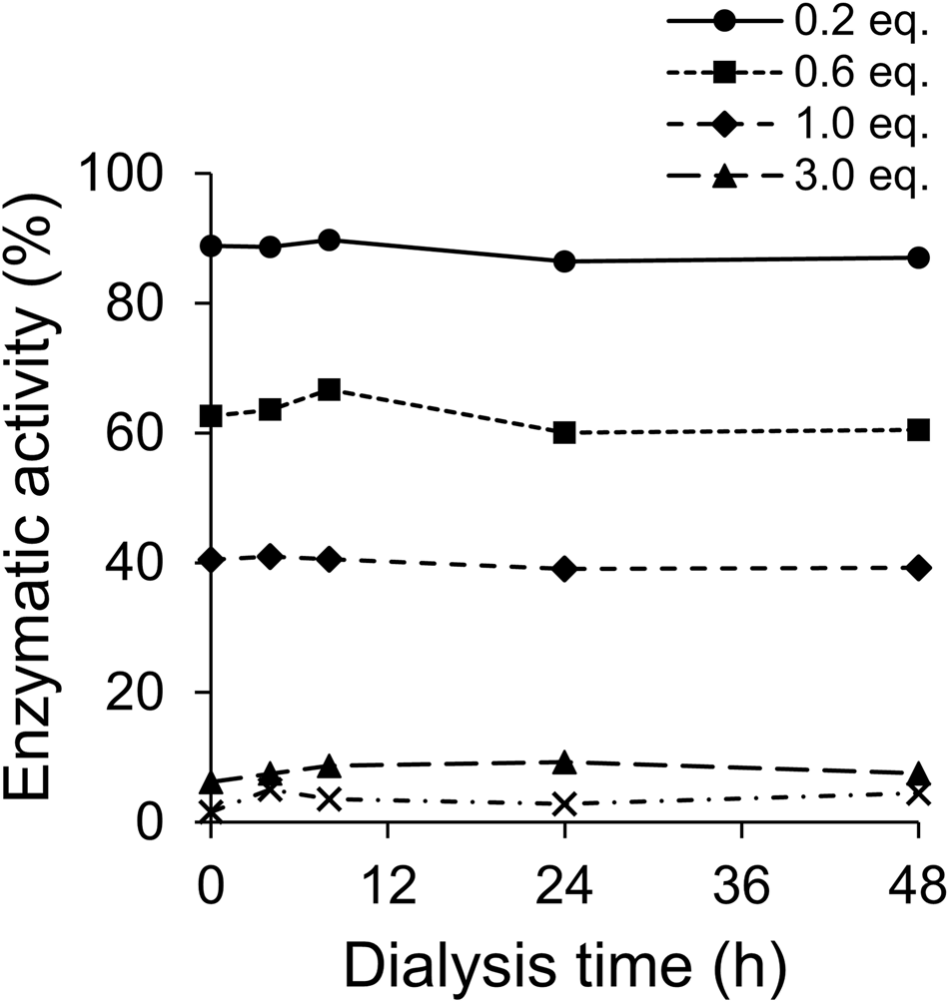
**Dialysis assay results of 2 with** ***h*OAT**

To determine the irreversibility of *h*OAT inactivation by **2**, dialysis assays were conducted. *h*OAT was first incubated with **2** at molar ratios of 0.2, 0.6, 1.0, 3.0, and 10 equivalents overnight, and it was confirmed that the pre-inactivated *h*OAT samples retained approximately 0–90% of their initial activity (Dialysis time = 0 h in Fig. 3), depending on the treated equivalents. The pre-inactivated samples and vehicle-treated *h*OAT control were then dialyzed in PLP-containing buffer to monitor potential recovery of enzymatic activity. As a result, no significant recovery in enzyme activity was observed for any of the samples after 48 h of dialysis (Fig. 3), indicating that **2** inactivates *h*OAT by irreversibly binding to the active site.

**Scheme 3:**
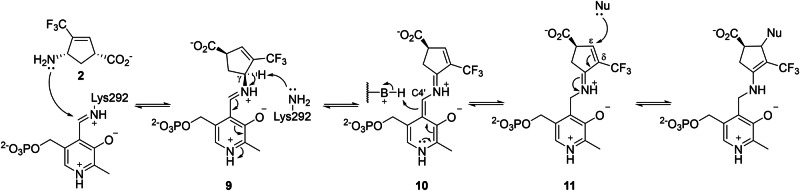
**Initially proposed inactivation mechanism of OAT by 2**

Based on the irreversible binding indicated by the dialysis assay, and inspired by previously reported mechanisms of OAT inactivators that covalently bind to OAT [18**–**21, 29, 30], the inactivation pathway of **2** was initially hypothesized as depicted in Scheme 3. Upon binding to the *h*OAT active site, **2** was proposed to form the corresponding external aldimine (**9**). Assuming that **9** undergoes the same steps as the catalysis of ornithine (Scheme 2B), the intermediate is subjected to γ-deprotonation to form a quinonoid (**10**), and re-protonation at the C4’ position to yield an external ketimine species (**11**). At this stage, conjugation of the ketimine with the δ-ε double bond was expected to generate a Michael acceptor, enabling nucleophilic attack and consequent covalent bond formation.

### Crystal structure of the final adduct of *h*OAT inactivated by 2

To validate the proposed mechanistic pathway, an X-ray crystal structure of *h*OAT complexed with **2** was obtained. *h*OAT was crystallized using a previously reported hanging-drop method [33]. Holoenzyme crystals were grown over 4 days prior to being soaked with an excess of **2** and αKG for 24 h. Following the 24 h soaking duration, crystals, which diffracted to 1.93 Å resolution, were frozen. The structure of *h*OAT, soaked with **2** and αKG, was solved with the use of a monomer from the previously published *h*OAT structure (PDB 6V8C) as the starting model for molecular replacement. Following model building and refinement cycles, the refined molecular model contained three monomers in one asymmetric unit in the *P*3_2_21 space group with *R*_free_/*R*_work_ values of 20.17/18.67% (Table S1).

**Fig. 4:**
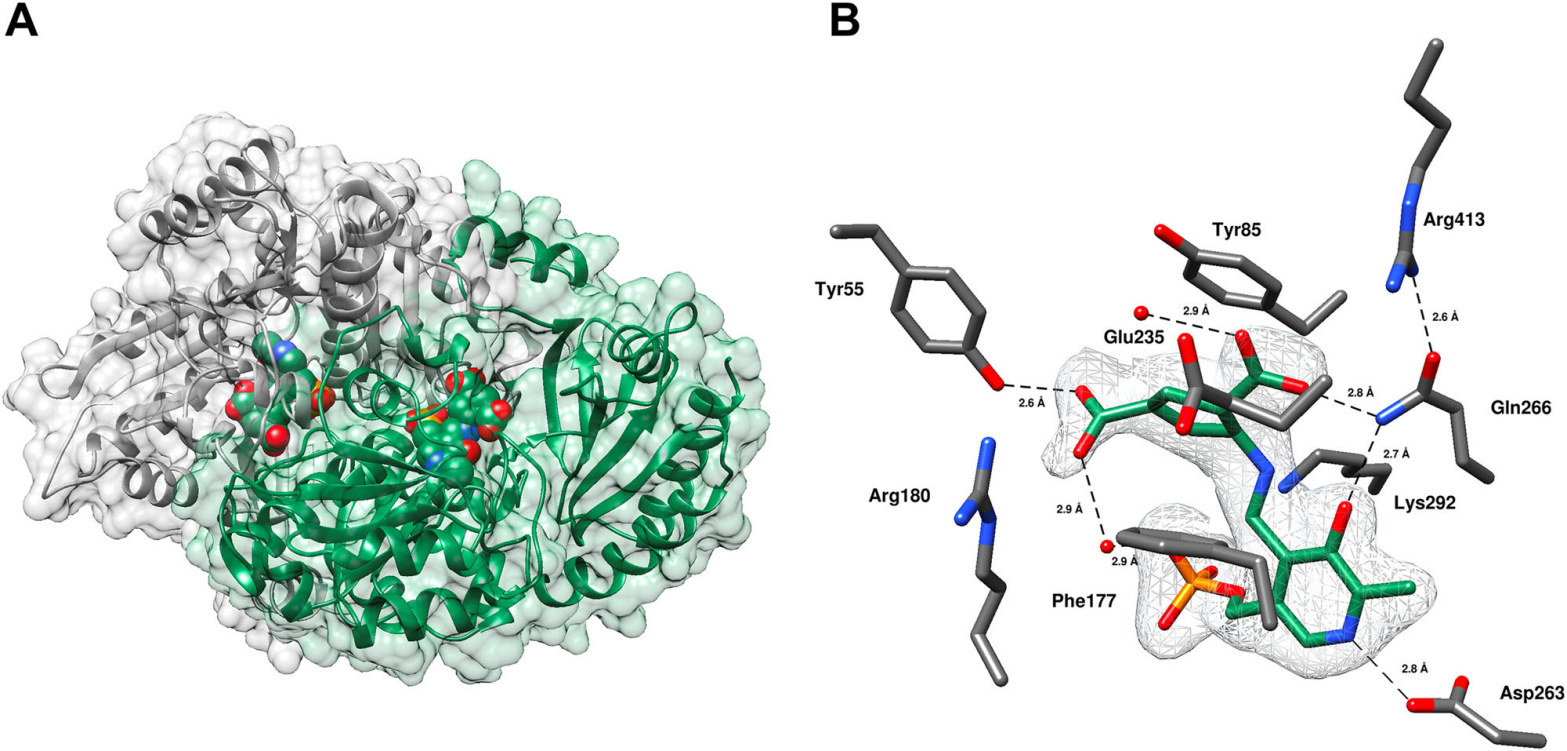
**Crystal structure of the proposed final adduct of** ***h*OAT inactivated by 2 (PDB 10LW)**. (**A**) Overall view of the refined *h*OAT dimer with the molecular surface shown; ligand atoms are displayed in CPK representation. (**B**) Close-up view of the active site with the detected ligand and surrounding residues shown as sticks (ligand in green, residues in gray). The polder map (*F*_o_-*F*_c_) is at 4.1 *σ*

The 24 h soaking duration was hypothesized to be sufficient to capture the final adduct of the reaction with *h*OAT; however, the resulting electron density in the active site revealed a structure that contradicted the initially proposed inactivation mechanism (Scheme 3), which includes nucleophilic addition at the ε-position. Instead of a substituent at the ε-position of the final adduct, the structure showed a structural change at the δ-position (Fig. 4). The lack of tetrahedral density at the δ-moiety indicated that at least one fluorine of the original trifluoromethyl of **2** has been eliminated from this position. The Lys292 side chain is pointed slightly downward, avoiding interaction with any nearby active site residues. While Tyr55 maintains a strong hydrogen bonding interaction (2.6 Å) with one oxygen of the α-carboxylate of **2**, Arg180 does not participate in any direct or indirect interactions with the inactivator. Rather, the other α-carboxylate oxygen interacts indirectly with a phosphate oxygen of PLP through an ordered water molecule. The cyclopentene scaffold of **2** does not stretch as far across the binding pocket as cyclohexene scaffold inactivators of *h*OAT [29, 30, 33], possibly accounting for the lack of interaction with Arg180 (Fig. 4).

**Fig. 5:**
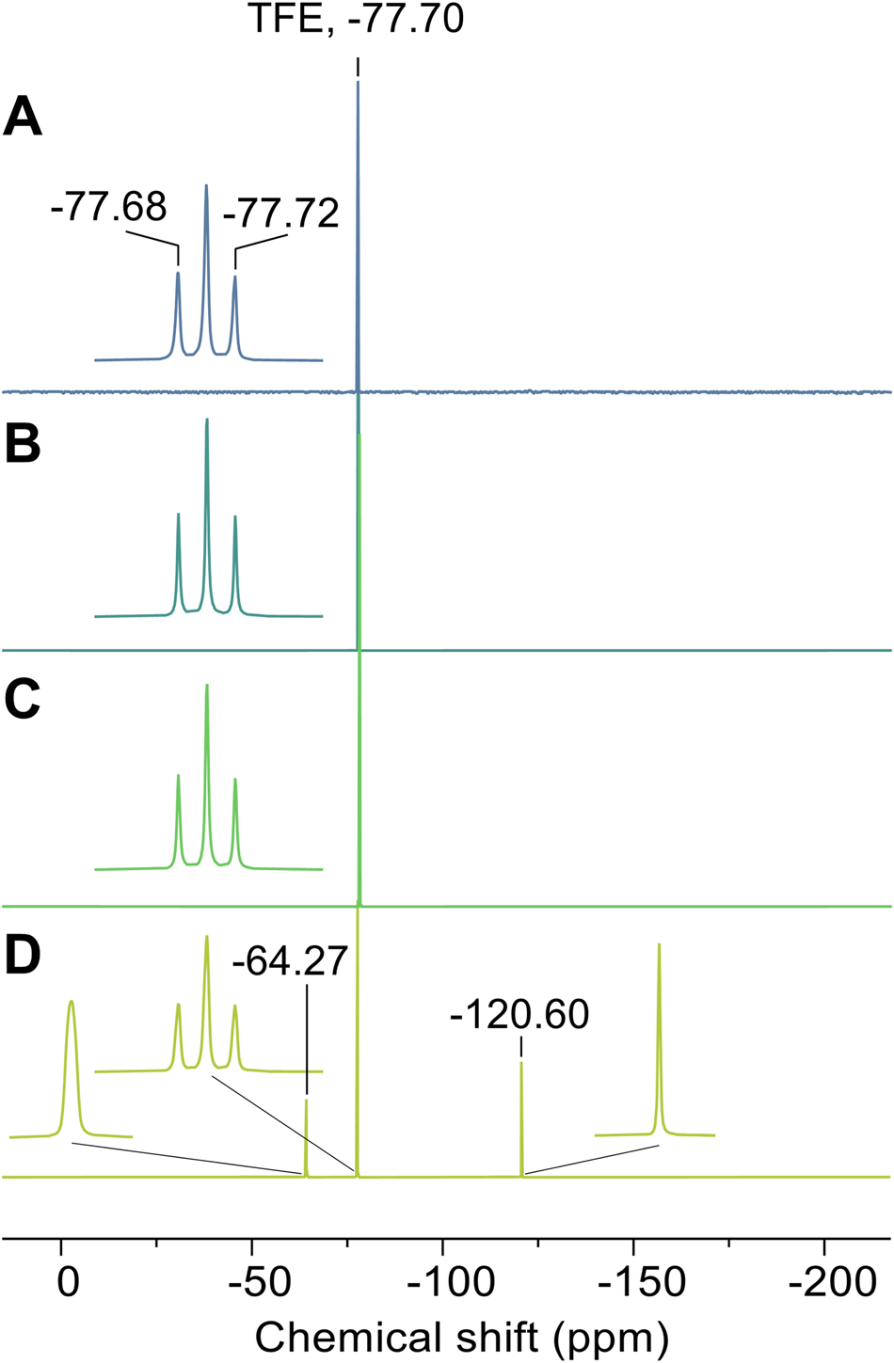
^**19**^**F NMR results**. ^19^F NMR spectra of (**A**) vehicle-treated, unmodified *h*OAT, (**B**) **2**-treated *h*OAT, (**C**) denatured *h*OAT after treatment of **2**, and (**D**) filtrate of *h*OAT–**2** reaction mixture. TFE, 2,2,2-trifluoroethanol

Considering fluoride release, the δ-moiety observed in the crystal structure could be a difluoromethylenyl or a fully hydrolyzed carboxylate group, as reported in the crystal structures of **1** in GABA-AT [23] and OAT [21]. To distinguish between these possibilities, *h*OAT was pre-incubated with **2** and washed by centrifugal filtration with D_2_O before being subjected to ^19^F nuclear magnetic resonance spectroscopy (^19^F NMR). Comparison of the ^19^F NMR spectrum of pre-incubated *h*OAT with that of a vehicle-treated control enzyme indicated no remaining fluorine atoms in the final adduct of **2** (Fig. 5). Aside from the signals for the internal standard (2,2,2-trifluoroethanol; –77.70 ppm relative to CFCl_3_), no additional fluorine peaks were detected, even after denaturation of ~20 μM enzyme, whereas the filtrate from the *h*OAT–**2** reaction mixture displayed singlet signals at –64.27 ppm, which falls within the range for trifluoromethyl groups (between –60 and –80 ppm) of unreacted **2**, and −120.60 ppm for released fluoride ions. Thus, the ^19^F NMR spectra indicate that the final adduct formed by treatment of *h*OAT with **2** no longer contains fluorine at the δ-position, and the δ-moiety possibly is a hydrolyzed carboxylate.

### Observation of a stable quinonoid intermediate by UV-Vis spectroscopy

**Fig. 6:**
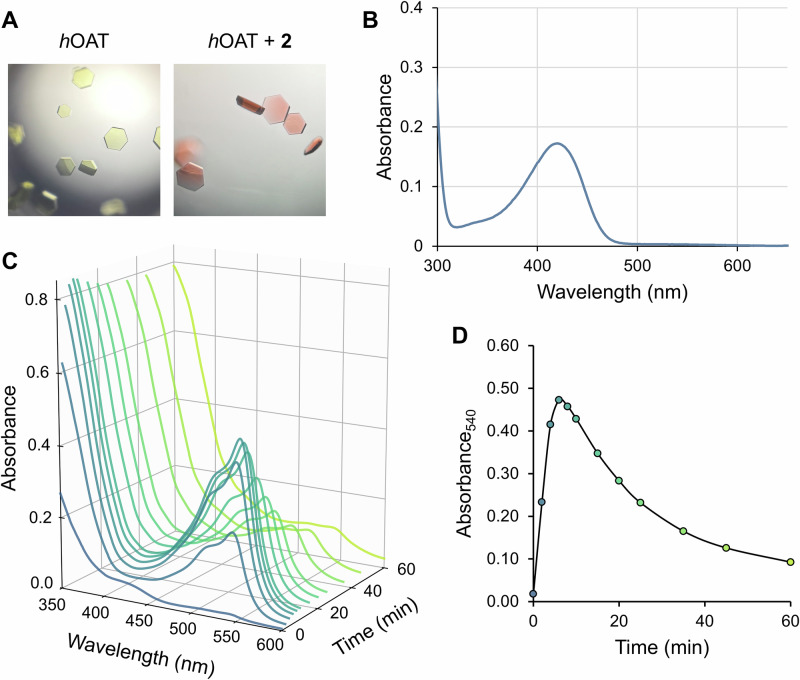
**Detection of quinonoid intermediate by UV-Vis spectroscopy with** ***h*OAT**. (**A**) Color change of *h*OAT crystal after treatment of **2** in the presence of αKG. (**B**) UV-Vis spectrum of untreated, PLP-bound *h*OAT. (**C,** **D**) Changes in UV-Vis spectrum of *h*OAT from the time of treatment with **2** and αKG (**C**) and its absorbance at 540 nm wavelength, which corresponds to a quinonoid species (**D**)

During the crystal-soaking, a notable color change of the *h*OAT crystals to a magenta-based color was observed upon incubation with **2** and αKG (Fig. 6A), and the crystals returned to a dull yellow, almost colorless after 24 h. The *h*OAT holoenzyme crystals with PLP in the active site exhibit a bright yellow color, which is known to demonstrate an absorption peak around 420–430 nm [34]. Typically, *h*OAT crystals become nearly colorless upon treatment with many inactivators that rapidly consume the PLP, based on their high *k*_inact_. In contrast, this bathochromic shift toward magenta-based color by treating **2** suggests the formation of a species with a larger conjugation system than PLP or PMP (peak absorption at ~330–340 nm) [35]. Thus, the formation of a long-lasting, stable quinonoid rather than a normal transient quinonoid intermediate has been hypothesized, as quinonoid species were known to have absorbance in the 500–570 nm range [35].

**Scheme 4:**
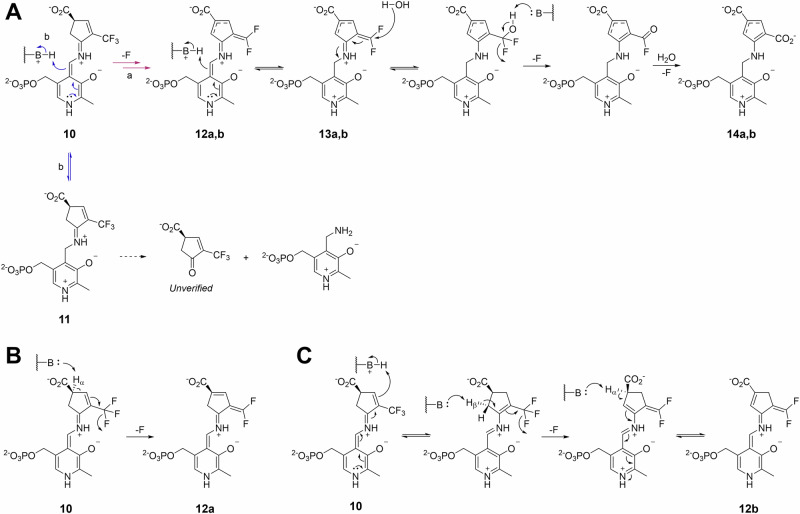
**The most consistent inactivation mechanism of OAT by 2 with experimental evidence (pathway a) and proposed turnover mechanism (pathway b)**. (**A**) The entire pathways involving the possible stable quinonoids (**12a** and **12b**) and the following stages. (**B,** **C**) Possible pathways leading to the stable quinonoid include (**B**) direct α-deprotonation of the initial quinonoid (**10**) and (**C**) consecutive deprotonation followed by re-protonation

To further investigate the color change observed during crystal soaking with **2** and αKG, UV-Vis spectroscopy was conducted. Prior to the inactivator treatment, a spectrum of the *h*OAT holoenzyme was collected, exhibiting an absorption peak at ~420 nm (Fig. 6B), consistent with that of the reported internal aldimine species [33]. Upon incubation of *h*OAT with **2** in the presence of αKG, a new species with an absorption at ~540 nm progressively accumulated, reaching maximum absorbance at around 6 min (Fig. 6C and D). However, when *h*OAT was treated with **2** in the absence of αKG, no significant accumulation of the 540 nm species was detected; instead, the peak near 340 nm, corresponding to a PMP species, increased rapidly, and remained throughout the observation period (Figure S1). This is because *h*OAT converts PLP to PMP with the formation of an aldehyde or ketone turnover product and requires PLP regeneration using αKG (Scheme 1) [3]; thus, only a minimal proportion of the inactivation pathway can proceed without αKG. Therefore, the UV-Vis results indicate that the turnover pathway of **2** does not form a stable quinonoid. **2** undergoes a bifurcating step to define two distinct pathways: (1) an inactivation pathway involving the formation of a relatively stable quinonoid intermediate (**12a** and **12b** in pathway a; Scheme 4A), and (2) a turnover pathway that yields a ketone turnover product and PMP without formation of the stable quinonoid intermediate (pathway b; in Scheme 4A, an unverified possible turnover pathway was suggested). To generate a stable quinonoid, the first transient quinonoid **10** can be directly deprotonated at its α-position, yielding a stable quinonoid **12a** (Scheme 4B). Also, **10** is expected to enable re-protonation at the ε-position of the inactivator, because the PLP ring and the inactivator ring constitute an extended, fully conjugated system to the ε-position (Scheme 4C). A subsequent series of deprotonations at the β- and α-positions would then be accompanied by fluoride elimination at the δ-trifluoromethyl group, similar to an isoxazole adduct generation by d-cycloserine with d-amino acid aminotransferase [36]. As a result, the pathway revisits a quinonoid species with a δ-difluoromethylenyl group (**12a** and **12b**), which can undergo C4’ re-protonation to yield an external ketimine (**13a** and **13b**) followed by hydrolysis of the δ-difluoromethylenyl group, in a similar manner to the inactivation mechanisms reported for CPP-115 with GABA-AT [23], ultimately yielding a dicarboxylate adduct (**14a** and **14b**).

### Crystal structure of the stable quinonoid intermediate in *h*OAT

To capture the structure of the stable quinonoid intermediate in *h*OAT, crystal soaking was employed. Holoenzyme crystals were grown for 7 days prior to being soaked with **2** and αKG, observing crystals shift to a bright, magenta (Fig. 6A), before being immediately looped and frozen. The crystals diffracted to 1.83 Å resolution, and the structure was solved utilizing a monomer from the previously published structure of *h*OAT (PDB 8V9M) as the starting model for molecular replacement. Following model building and refinement cycles, the refined molecular model contained three monomers in one asymmetric unit in the *P*3_2_21 space group with *R*_free_/*R*_work_ values of 22.71/19.14% (Table S1).

**Fig. 7:**
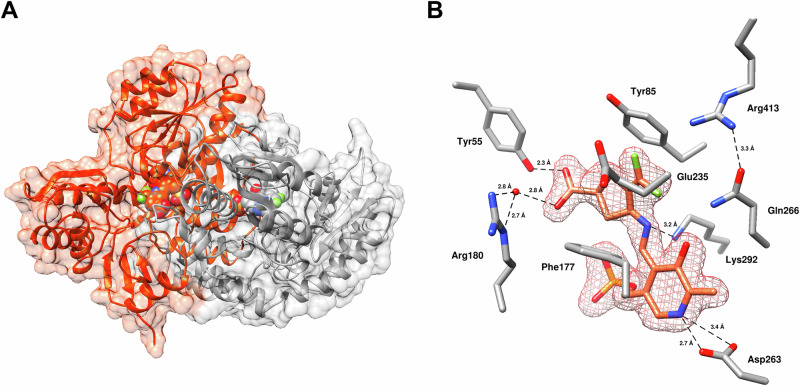
**Crystal structure of the stable quinonoid intermediate in** ***h*OAT (PDB 10LX)**. (**A**) Overall view of the refined *h*OAT dimer with the molecular surface shown; ligand atoms are displayed in CPK representation. (**B**) Close-up view of the active site with the quinonoid intermediate and surrounding residues shown as sticks (ligand in orange, residues in gray). The polder map (*F*_o_-*F*_c_) is at 4.1 *σ*

The electron density in the active site indicated a quinonoid intermediate (Fig. 7), consistent with the suggested structures for the stable quinonoid (**12a** and **12b**). Specifically, polder map density indicates that the inactivator moiety and the pyridine ring of PLP are present within the same plane, and the C4-C4’ bond of PLP exhibits planar density. Based on the lack of observed tetrahedral electron density at the δ-moiety, at least one fluorine of the original trifluoromethyl substituent has been eliminated. Therefore, a difluoromethylene was built at the δ-position, which appears to maintain sp^2^ geometry. Additionally, the distance between the δ-moiety and Gln266 was > 4.5 Å, whereas the δ-moiety of the final adduct was forming a hydrogen bonding interaction with the side chain of Gln266 (2.8 Å) (Fig. 4B). The α-carboxylate of **2** forms a strong hydrogen bond (2.3 Å) with Tyr55, which recognizes an α-amino group of ornithine [37], as well as an indirect hydrogen bonding interaction with Arg180 through an ordered water, possibly contributing to the recognition of **2** by *h*OAT. Similar to the final adduct proposed in Fig. 4, the salt bridge between Glu235 and Arg413 is disrupted, with Glu235 turning away from the active site of *h*OAT entirely.

Highly stable quinonoid intermediates may be attributed to the use of an alternative substrate or inactivator in the binding pocket as well as the difficulty of the C4’ position of PLP to be re-protonated. From a structural perspective, the 3.2 Å hydrogen bonding interaction between Lys292 and the imine nitrogen of PLP may render a proton of Lys292 unavailable for re-protonation of the C4’ position. Therefore, the inactivator-PLP complex accumulates in the quinonoid intermediate state until re-protonation can occur.

Thus, other than the formation of the initial transient quinonoid (**10**; Scheme 3), **2** is most likely converted into another relatively stable quinonoid intermediate (**12a** and **12b**; Scheme 4) in the *h*OAT active site, thereby yielding the cyclopentadiene dicarboxylate final adduct (**14a** and **14b**; Scheme 4) to inactivate the enzyme. The UV-Vis spectra in the absence of αKG indicate that the turnover pathway does not involve the accumulation of a quinonoid species, implying that this pathway must branch off before the stable quinonoid formation. Consequently, the turnover pathway may proceed via re-protonation at the C4’ position of the first transient quinonoid to form the external ketimine (**11**; Scheme 3), rather than via re-protonation at the ε-position to form the stable quinonoid. Further studies will be required to more clearly define the detailed steps and branching point of the turnover pathway. Also, to leverage the mechanistic insight of this OAT inactivator for enhancing its selectivity against GABA-AT, a similar mechanistic investigation should be conducted for GABA-AT and compared to the mechanism in this study.

### Possible designs of OAT inactivators from 2

Since the crystal structure of the final adduct showed that it cannot form an interaction with Arg180, the ring expansion strategy [29] might enhance its activity with OAT by improving the binding affinity. Additionally, the 6-membered ring may exceed the size of the smaller active site of GABA-AT, thereby enhancing selectivity against GABA-AT. However, in this case, the position and numbers of endocyclic double bonds must be carefully modulated because they can affect the electronic properties of the ring and the pKa of the α- and β-protons, which are likely extracted during the inactivation pathway. If the α- or β-deprotonation is a rate-limiting step of inactivation by **2**, additional electron-withdrawing groups, such as fluorine, at the inactivator ring may reduce their pKa to accelerate the inactivation pathway.

## Conclusion

OAT participates in glutamine metabolism, which is upregulated to support cancer cell proliferation [4], and its gene expression is regulated by β-catenin signaling, which is aberrantly activated in many cancer cells [12]. Taken together, OAT has been recognized as an emerging therapeutic target for HCC [1]. In previous work, we identified *h*OAT inactivators and showed that they suppress the proliferation of HCC cells in vitro [14], supporting the potential of OAT inactivation as an anticancer strategy. In this study, the relatively less characterized compound among these inactivators, (1*R*,4*S*)-4-amino-3-(trifluoromethyl)cyclopent-2-ene-1-carboxylic acid (**2**), was evaluated as an OAT inactivator by time-dependent inactivation assays with *h*OAT. The resulting kinetic profiles revealed that **2** is a moderate OAT inactivator with favorable OAT/GABA-AT selectivity. On these findings, the inactivation mechanism of **2** toward OAT was investigated using dialysis experiments, X-ray crystallography, and UV-Vis spectroscopy. The crystal structure of 24-h soaked *h*OAT crystals indicated that the final adduct, in combination with the ^19^F NMR data, most likely contains a dicarboxylate. Notably, UV-Vis and short-soaking crystallography indicated the presence of a relatively stable quinonoid intermediate. Thus, the suggested inactivation mechanism, according to the results in this study, includes the formation of a stable quinonoid (**12a** and **12b**) in the *h*OAT active site. To the best of our knowledge, inactivation pathways via transient quinonoid intermediates have previously been reported for OAT inactivators, but none observed to be as stable as **12**, while stabilized quinonoid intermediates have reported in other PLP-dependent enzymes, such as tyrosine phenol-lyase [38] and tryptophan indole-lyase [39]. In conclusion, the mechanism proposed in this study may provide a basis for further enhancing the OAT inactivation efficiency of **2** and could also inform the rational design of new inactivators that exploit distinct mechanistic features. If further investigation regarding the GABA-AT inactivation mechanism of the compound is conducted in the future, the mechanistic and/or structural differences between the two pathways can be employed to enhance its selectivity toward OAT.

## Materials and methods

### Synthesis of (1*R*,4*S*)-4-amino-3-(trifluoromethyl)cyclopent-2-ene-1-carboxylic acid (2)

All reagents and solvents were obtained from commercial suppliers and used without further purification. ^1^H and ^13^C nuclear magnetic resonance (NMR) spectra were recorded on a Bruker Avance III spectrometer (Bruker; Billerica, MA, USA) operating at frequencies of 500 MHz and 126 MHz, respectively. The spectra were acquired in CD_3_OD. Chemical shifts and coupling constants are reported in parts per million (δ) and Hz, respectively. The spectral data were presented as follows: chemical shift, multiplicity (s, singlet; d, doublet; t, triplet; q, quartet; dd, doublet of doublet; dt, doublet of triplet; m, multiplet), coupling constant, and integration. Mass spectra were obtained using a Thermo TSQ Quantum system (Thermo Fisher Scientific; Waltham, MA, USA) in the positive ion mode using atmospheric pressure chemical ionization (APCI) with the Agilent Infinity 1260 HPLC system (Agilent Technologies; Santa Clara, CA, USA) with the following conditions: Phenomenex Kintex C-18 column (50 × 2.1 mm, 2.6 μm); mobile phase, 5–100% acetonitrile/water containing 0.05% trifluoroacetic acid at a flow rate of 0.9 mL/min for 6 min. (1*R*,4*S*)-4-amino-3-(trifluoromethyl)cyclopent-2-ene-1-carboxylic acid (**2**) was synthesized according to the previously reported synthetic procedure [27] and isolated as a white solid by crystallization in acetonitrile. The identity and purity of **2** were confirmed by ^1^H NMR, ^13^C NMR, and mass spectrometry, and all data were in agreement with the reported values. ^1^H NMR (500 MHz, CD_3_OD) δ 6.93 (s, 1H), 4.60 (dd, *J* = 8.8, 4.9 Hz, 1H), 3.94 – 3.85 (m, 1H), 2.87 (dt, *J* = 14.4, 8.8 Hz, 1H), 2.34 (dt, *J* = 14.4, 5.1 Hz, 1H). ^13^C NMR (126 MHz, CD_3_OD) δ 173.89, 143.88 (t, *J* = 4.8 Hz), 132.11 (q, *J* = 34.0 Hz), 123.09 (q, *J* = 269.7 Hz), 54.71, 49.92, 33.45. MS (ESI): [M + H]^+^ = 195.94.

### Enzymes and assays

Recombinant *h*OAT [31] and pyrroline-5-carboxylate reductase 1 (PYCR1) [40] were prepared according to previously described expression and purification protocols without further modification. *h*OAT activity was measured using a continuous coupled assay based on the PYCR1-dependent reduction of P5C, as described in our previous work [32]. Briefly, reactions were carried out at 37 °C in 100 mM potassium pyrophosphate buffer (pH 8.0) containing 0.025 mM PLP, 10 mM αKG, 0.4 mM reduced form of nicotinamide adenine dinucleotide (NADH), recombinant human PYCR1, and ornithine. The reactions were monitored using a Synergy H1 hybrid multimode microplate reader (BioTek, USA), by measuring the decrease in absorbance at 340 nm for NADH consumption during the reaction of P5C with PYCR1. For inactivation assays, *h*OAT was pre-incubated with the test compound under the indicated conditions, and initial rates were obtained from the linear portion of the progress curves as described in the reported procedures.

### Crystallization

Holoenzyme *h*OAT was exchanged into a buffer containing 50 mM Tris, 200 mM NaCl, and 100 µM PLP (pH 7.4) using a 50,000 Da molecular weight cutoff (MWCO) Amicon^®^ Ultra centrifugal filter (MilliporeSigma, USA). The sample was then concentrated to ~5 mg mL^−1^. The hanging-drop vapor diffusion method was utilized to grow crystals containing 2 μL *h*OAT and 2 μL of well solution. Crystals were grown at 25 °C for 4 days, and the crystals with the best morphology appeared in the well solution containing 7.25–8.50% PEG 6000, 100–250 mM NaCl, 2.0–3.5% glycerol, and 100 mM Tricine (pH 7.8). Selected crystals with the best morphology were transferred to a crystal drop containing well solution supplemented with 30% glycerol, 10 mM αKG, and 10 mM of **2**. Crystals were soaked for 24 h or until appearing red in color, characteristic of the quinonoid intermediate, prior to being flash-cooled in liquid nitrogen. For the short-duration crystal soak to capture the quinonoid intermediate, monochromatic X-ray diffraction data were collected at the LS-CAT, Advanced Photon Source (APS), beamline 21-ID-D at Argonne National Laboratory (ANL, Argonne, IL). Data were collected at a wavelength of 1.127 Å and a temperature of 100 K using a Dectris Eiger 9 M detector. For the 24-hour crystal soak, monochromatic X-ray diffraction data were collected at LS-CAT, beamline 21-ID-G. Data were collected at a wavelength of 0.97856 Å and a temperature of 100 K using a Dectris PILATUS 6 M detector. All data sets were processed and analyzed with autoPROC [41]. The structure of OAT was solved by molecular replacement utilizing PHASER [42] in Phenix [43]. The search model was of the previously published structures of OAT (PDB code: 6V8C). The model building and refinement were performed utilizing Coot [44] and Phenix, respectively, until the lowest possible Rfree/Rwork factor values were achieved. UCSF Chimera [45] was used for the preparation of the structural depiction figures. The structure factors and refined model were deposited in the Protein Data Bank (Table S1).

### UV-Vis spectroscopy

A Shimadzu UV-2450 spectrophotometer was utilized to collect absorbance spectra from 700 to 250 nm at 25 ℃. The instrument was baselined with 1 mM ⍺KG in a reaction buffer containing 50 mM HEPES pH 7.5 and 200 mM NaCl. The initial concentration of *h*OAT was determined with A280 measurement and the molar extinction coefficient previously calculated with amino acid composition. An initial scan of 20 µM *h*OAT was collected, and various scans of *h*OAT incubated with 10 mM 2 were collected both in the absence and presence of 1 mM ⍺KG.

### ^19^F NMR spectroscopy

The *h*OAT sample was D_2_O-washed with Amicon^®^ Ultra centrifugal filters after complete inactivation by treating excess **2** or after treating the same volume of vehicle for the unmodified control sample. Each sample was then diluted to ~20 μM. During washing with D_2_O, the first filtrate from the *h*OAT-**2** reaction mixture was collected. 2,2,2-Trifluoroethanol (analytical standard; MilliporeSigma) was added as an internal standard at a final concentration of 20 μM for each sample. Samples were run on a Bruker Neo 600 MHz system with a QCI (HFCN) Cryo Probe w/Z-Gradient at 298 K for 960 scans. For the denatured sample, after the measurement, the **2**-treated *h*OAT sample was heated to 55 °C with 10 equivalents of formic acid and measured under the same conditions.

#### Supplementary materials

Supplementary figures and tables, ^1^H and ^13^C spectra, and crystallographic data.

#### Accession codes

Atomic coordinates and corresponding structure factors for the two crystal-soaking complexes of *h*OAT with **2** have been deposited in the Protein Data Bank (PDB) as 10LW and 10LX. Authors will release the atomic coordinates upon article publication.

## Supplementary information

Supplementary Materials

## Data Availability

No datasets were generated or analysed during the current study.
