# Supplementary material for: Inactivation of ornithine aminotransferase by (1R,4S)-4-Amino-3-(trifluoromethyl)cyclopent-2-ene-1-carboxylic acid via a stable quinonoid intermediate
Source: Med Chem Res. 2026 Mar 15;35(4):792–803. doi: 10.1007/s00044-026-03538-1 (PMC13128779; doi:10.1007/s00044-026-03538-1)

Supplementary Materials for

Inactivation of Ornithine Aminotransferase by (1*R*,4*S*)-4-Amino-3-(trifluoromethyl)cyclopent-2-ene-1-carboxylic Acid via a Stable Quinonoid Intermediate

Koon Mook Kang[1,2,†], Abigail L. Vargas[3,†], Wei Zhu[1,2,§], Inna Sokolenko[1,2], Dali Liu[3], Richard B. Silverman[,1,2,4,5,6]*

[1] Department of Chemistry, Northwestern University, Evanston, Illinois 60208, United States

[2] Chemistry of Life Processes Institute, Northwestern University, Evanston, Illinois 60208, United States

[3] Department of Chemistry and Biochemistry, Loyola University Chicago, Chicago, Illinois 60660, United States

[4] Department of Molecular Biosciences, Northwestern University, Evanston, Illinois 60208, United States

[5] Center for Developmental Therapeutics, Northwestern University, Evanston, Illinois 60208, United States

[6] Department of Pharmacology, Feinberg School of Medicine, Northwestern University, Chicago, Illinois, 60611, United States

[†] These authors contributed equally.

* Corresponding author:

✉ Richard B. Silverman
Agman@chem.northwestern.edu

[§] Current address: Insilico Medicine Shanghai Ltd., Shanghai 201203, China

Supplementary Figures and Tables

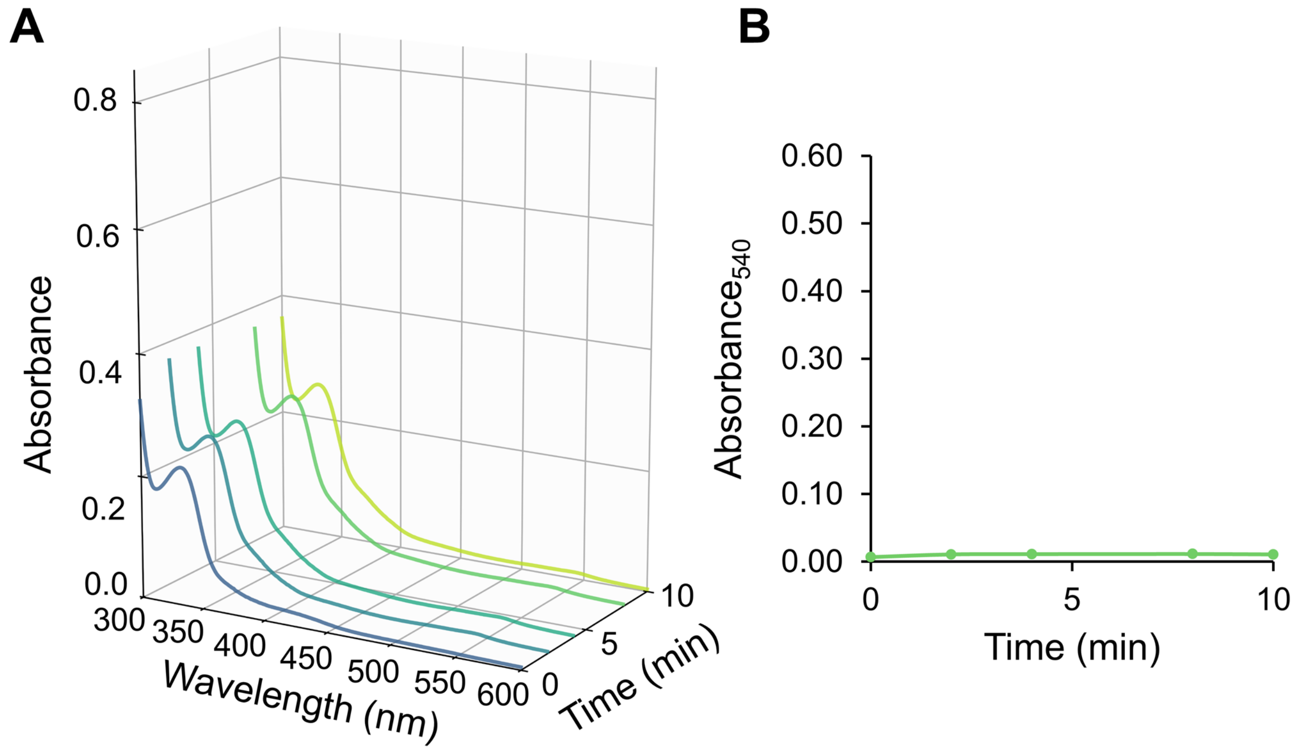

Figure S1. UV-Vis spectroscopy results of *h*OAT in the absence of αKG. Changes of UV-Vis spectrum of *h*OAT by the time from the treatment of **2** (**A**) and its absorbance at 540 nm wavelength (**B**).

Table S1. Data collection and refinement statistics

Crystal Complex	24-hour Soaking Final Adduct	Short-Soaking for Quinonoid Capture
Ligand	**2**	**2**
PDB Code	10LW	10LX
**Data Processing**		
Wavelength (Å)	0.97872	0.920105
Resolution range (Å)	50.01-1.93	34.20-1.83
Space group	P 32 2 1	P 32 2 1
Unit cell dimensions (Å)	115.485, 115.485, 187.081	115.52, 115.52, 187.44
Unit cell angles (°)	90.0, 90.0, 120.0	90.0, 90.0, 120.0
Total reflections	707805	2667123
Unique reflections	102426	128275
Multiplicity	6.9 (6.8)	20.8 (19.0)
Completeness (%)	93.70 (96.62)	99.98 (99.88)
Mean I/Sigma(I)	16.8 (0.8)	12.7 (1.2)
Wilson B-factor	40.00	25.50
$^{b}R_{pim}$	0.027 (0.880)	0.048 (0.727)
$^{c}CC_{1/2}$	1.00 (0.329)	0.999 (0.322)
**Refinement**		
$^{d}R_{work}$	0.1797 (0.3319)	0.1990 (0.3156)
$^{e}R_{free}$	0.2184 (0.3851)	0.2339 (0.3230)
Number of Non-Hydrogen Atoms	10061	10444
Macromolecules	9464	9483
Ligands	111	81
Solvent	486	880
^{f}RMS(bonds) (Å)	0.007	0.014
RMS(angles) (°)	0.86	1.05
Ramachandran favored (%)	94.86	95.11
Ramachandran allowed (%)	4.89	4.73
Ramachandran outliers (%)	0.25	0.17
Average B-factor (Å^2)	51.89	33.95
Macromolecules (Å^2)	51.75	31.53
Ligands (Å^2)	56.01	31.27
Solvent (Å^2)	53.76	35.11

[a]The values for the highest-resolution bin are in parentheses, [b]Precision-indicating merging R, [c]Pearson correlation coefficient of two "half" data sets, $^{d}R_{work} = \Sigma|F_{obs} - F_{calc}|/\Sigma F_{obs}$, [e]Five percent of the reflection data were selected at random as a test set, and only these data were used to calculate R_{free}, [f]Root-mean square deviation.

Supplementary ^{1}H and ^{13}C NMR spectra

(1*R*,4*S*)-4-amino-3-(trifluoromethyl)cyclopent-2-ene-1-carboxylic acid (**2**)

^{1}H NMR spectrum (CD_3OD)

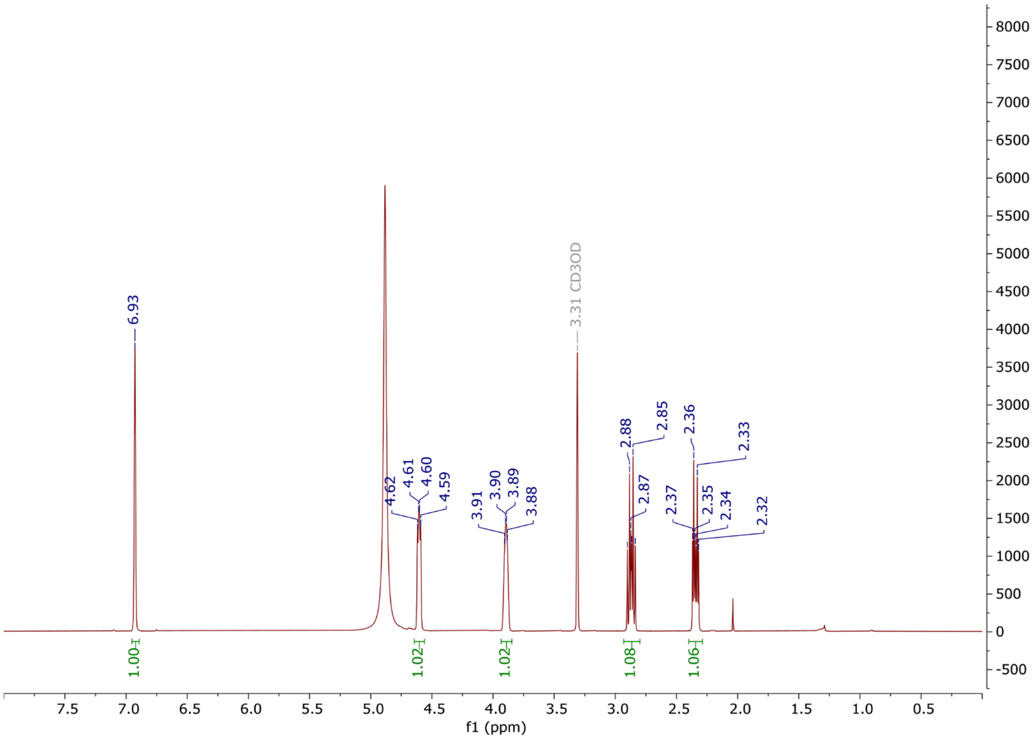

^{13}C NMR spectrum (CD_3OD)

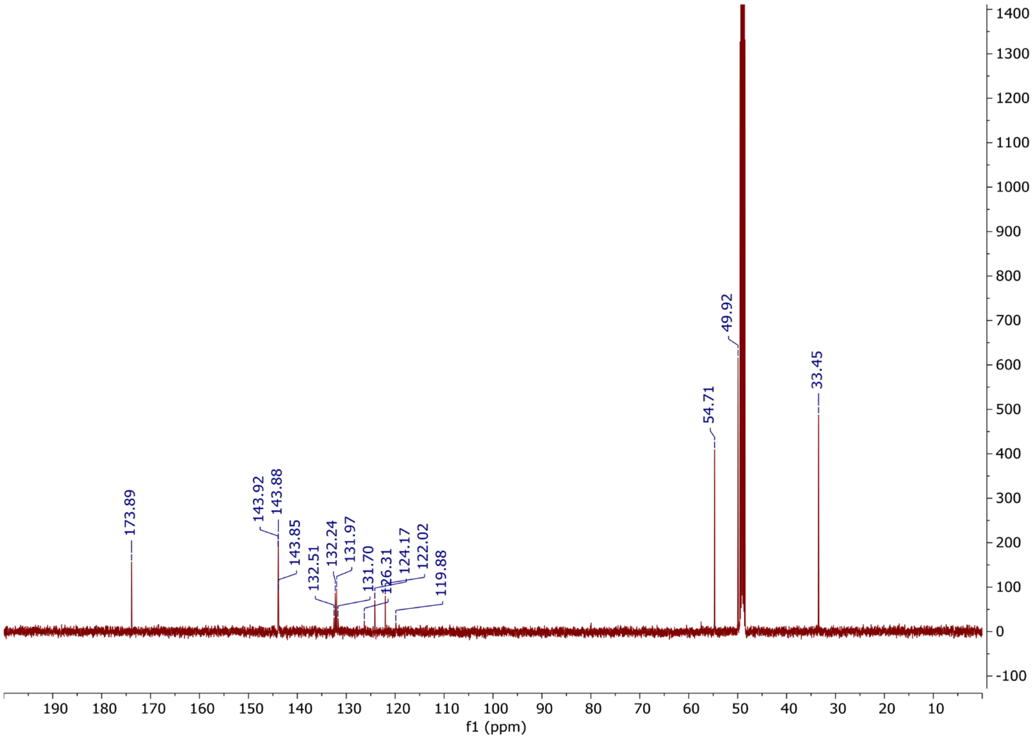

Supplement: Supplementary file 1 — Supplementary Materials [file 44_2026_3538_MOESM1_ESM.pdf]
